# Reducing 48-h emergency department revisits and subsequent admissions: a retrospective study of increased emergency medicine resident floor coverage

**DOI:** 10.1186/s12245-022-00471-z

**Published:** 2022-12-06

**Authors:** Pakhawadee Palungwachira, Gunnaree Montimanutt, Khrongwong Musikatavorn, Sorravit Savatmongkorngul

**Affiliations:** 1 Department of Emergency Medicine, Faculty of Medicine, Chulalongkorn University and King Chulalongkorn Memorial Hospital, Thai Red Cross Society, Bangkok, 10330 Thailand; 2 Department of Emergency Medicine, Faculty of Medicine, Ramathibodi Hospital, Mahidol University, Bangkok, 10400 Thailand

**Keywords:** Emergency department, Hospital admission, Revisit

## Abstract

**Background:**

Early unexpected hospital admission after emergency department (ED) discharge is an important topic regarding effective preventive measures. Reducing avoidable return visits can improve ED effectiveness and emergency care. This study evaluated the effects of an increase in the number of physicians and the 24-h coverage of emergency physicians on 48-h ED revisits with subsequent hospital admission. The characteristics and risk factors of the patients were also investigated.

**Results:**

This was a retrospective analysis performed 2 years before and 2 years after the implementation of an intervention in a tertiary care hospital in Thailand. The medical records of adult patients who revisited the ED within 48 h for related complaints were reviewed. The effect of the intervention was analyzed, and a prediction model was developed based on logistic regression.

After implementing the intervention, the hospital admission rate at the second ED visit decreased from 44.5 to 41.1%; no significant difference was found (95% confidence interval (CI) − 5.05 to 11.78). Patients who required hospital admission had a significantly higher comorbidity score, more ED visits, and more hospitalizations within the past 12 months. A significantly higher hospital admission rate was also observed among patients older than 60 years, those who had an initial infectious diagnosis, and those who had a higher triage severity level (ESI II) at their first visit. The odds ratio (OR) showed lower odds of hospital admission at the second visit in the postintervention period; this difference was not significant (OR 0.87; 95% CI 0.61 to 1.23).

**Conclusion:**

Our intervention did not significantly decrease the incidence of admission at an ED revisit. However, some factors identified in this study seem to have some benefits and might be helpful for preventing errors and constructing a standard discharge care plan for patients with these risk factors.

**Supplementary Information:**

The online version contains supplementary material available at 10.1186/s12245-022-00471-z.

## Background

In recent years, the roles of emergency departments (EDs) in many countries have increased in importance in relation to economic and clinical factors and changes in the health care system [1, 2]. As EDs play an essential role in the delivery of acute ambulatory and inpatient care, there has been an increase in the utilization of EDs in many countries [3, 4]. During the past decade, ED crowding has occurred and increased in prevalence [5]. The potential negative effects of ED overcrowding have been assessed, and several corrective measures have been proposed [5, 6]. A previous systematic review showed that unscheduled ED returns may be a quality indicator of higher resource utilization, treatment delays, and excessive mortality [7–9]. Other studies reported that the mortality rate of patients with revisit admissions was relatively high, ranging from 4.1 to 10% [10, 11], which may result in diagnostic or treatment delays or the early release of patients from the ED, resulting in ED revisits shortly after discharge [12]. 

Thailand is a middle-income country that adopted emergency medicine practice models from the United States with an advancing health care system that has different patient populations, settings, and instrument availability. To our knowledge, there have been few studies on unplanned revisits conducted in Thailand. One retrospective single-hospital study described the characteristics of the patients who revisited the ED [13]. Another study examined the factors associated with 48-h ED revisits that showed that misdiagnosis and suboptimal management contributed half of the repeat visits of ED patients [14]. No study has implemented an intervention to decrease the number of ED revisits.

A number of articles have documented the impact of overcrowding on the quality of care and medical errors [15]. The widely cited negative consequences of ED overcrowding are long waiting periods, patient discontent, overstressed health care professionals, and safety and efficiency problems [7, 16, 17]. Currently, more than half of patients admitted to the hospital in the United States start their hospital stay in the ED [18]. In addition to the important role of EDs in assessing and stabilizing seriously ill and injured patients, EDs support primary care practices by performing complex diagnostic workups and managing overflow, after-hours, and weekend demands for care. As a result of these shifts in practice, emergency physicians (EPs) are responsible for the major decisions of approximately half of all hospital admissions [1]. Maintaining a noncrowded ED to accommodate patients while also using it as a holding area prior to admission requires hospital and ED staff to speed up decision-making processes, including those related to discharges. As a means to reduce ED crowding and unscheduled related return visits, an intervention of assigning 24-h emergency medicine (EM) resident coverage along with increasing the number of EM residents per shift was implemented (Supplementary Figure S1).

Although the literature discussing ED revisits has mentioned unexpected consequences in ED-discharged patients [19, 20], no report has focused on events in which patients rapidly deteriorated after being discharged and were then admitted at return visits. One retrospective cross-sectional study demonstrated the utilization of fewer resources, without higher hospital admission or mortality rates, among patients with ED revisits within 72 h than among first-time ED visitors [21]. Indeed, studies focusing on this type of serious adverse event in ED-discharged patients are rare.

This present study was designed to investigate an intervention to reduce ED revisits and recognize patients at higher risk for a revisit and subsequent admission, which is suggested to be a more refined and reliable indicator [22]. An intervention characterized by an increase in the number and 24-h coverage of EM residents was implemented, as we believe that it could result in a reduction in the number of patients in this revisit population. This intervention was instituted within a 2-year period and was maintained thereafter.

## Methods

### Study design

This study was based at the ED of the urban 1500-bed tertiary care King Chulalongkorn Memorial University Hospital (KCMH), Bangkok, Thailand. The annual ED visit rate among adult patients is approximately 40,000 patients per year. The medical records of all adult patients who revisited the ED within 48 h after initial discharge from July 1, 2014, to June 30, 2019, were extracted from our ED administrative database and retrospectively reviewed in terms of patient information, disease category, length of stay during the first ED visit, hospital visits within the previous 12 months, underlying diseases, and triage level according to the emergency severity index (ESI) score. The change in the number of revisit-admission patients after our intervention was analyzed and compared along with the characteristics of the discharged and admitted patients in the second ED return visit. We used the Charlson comorbidity index to categorize comorbidities in our patients. The Institutional Review Board of our institution approved the protocol of the present study.

### Study population

Adult patients (> 15 years old) with ED unscheduled revisits within a 48-h period based on the time of the first visit discharge were included in the current study. Patients who revisited the ED for medical problems unrelated to the initial visits (Table 1) were excluded. The unscheduled related revisit patients were assessed manually by two Eps. If the categorization was inconsistent, the final decision was made by a third experienced EP who had considerable experience in identifying medical errors. Additional exclusion criteria included patients who had left the ED against medical advice.

**Table 1:** Definition of patients’ ED revisits

	Related to initial visits	Unrelated to initial visits
Scheduled revisits	A scheduled revisit for which the reason was related to the diagnosis of the initial visit	A scheduled revisit for which the reason was not related to the diagnosis of the initial visit
Unscheduled revisits	**An unscheduled revisit for which the reason was related to the diagnosis of the initial visit**	An unscheduled revisit for which the reason was not related to the diagnosis of the initial visit

Patients were divided into revisit and revisit-admission groups for further comparisons. The patients who were assigned to the revisit-admission group were the patients who had to be observed in the observation unit, admitted to the hospital, or referred to another hospital during the second visit. In the revisit-admission group, no patients died at their second visit.

### Interventions

The present study classified the nature of the return visits in accordance with the cause based on the method proposed in a previous study by Pierce et al. [23], with minor modifications. Cases were classified into 11 diagnostic groups by ICD 10 diagnosis, and the patients were categorized based on their first visit diagnosis.

The preintervention period was between July 1, 2014, and June 30, 2016. During that time, the ED area was staffed by full-time Eps of the KCMH and EM residents. Two EM residents worked during the morning shift and evening shift. One EM resident worked during a night shift but could do partial coverage for two weeks per month due to Thai EM resident training shift regulations. There were also rotating residents from other specialties who were assigned ED shifts and were available for the treatment of nonurgent patients. Please refer to Supplementary Figure S1 for the infographic information.

After the 1-year gap between July 1, 2016, and June 30, 2017, the corresponding postintervention patients during a period of 2 years were collected between July 1, 2017, and June 30, 2019. Both periods had the same number of Eps and coverage from rotating residents from other specialties. However, the increase in the number of EM residents was implemented to ensure 24-h ED coverage and more EM residents per shift. Three EM residents worked during the morning shift and evening shift. During the night shift, there were two EM residents in charge of full ED coverage for the whole month (Supplementary Figure S1). All doctors who have a Thai medical license have the authority to discharge patients themselves.

### Sample size and statistical analysis

We estimated that 2 years of sampling during each period (corresponding to approximately 500 revisit patients over 4 years) would allow for the reliable estimation of the incidence of subsequent admission in each period and each group of patients. The baseline incidence of unscheduled related return visits with subsequent admission from the hospital’s ED was 20%. A sample size of 237 patients from each group was sufficient to ensure an 85% power to detect a decrease of 10% in the incidence between the preintervention and postintervention periods. We omitted cases with missing data, which were 3.7% of cases, and analyzed the remaining data.

We compared comorbidities and severity by triage in relation to the need for admission at the second visit in groups according to the study period, age, sex, initial diagnosis category, length of stay, number of ED visits, and hospitalization within the past 12 months using 95% confidence intervals (CIs) for differences in proportions. Logistic regression was used in the case–control subsamples to determine the independent effect of our intervention or other risk factors on patients who required admission at their second visit while controlling for other potential confounders. The details for each diagnosis were recorded during the data collection. The analyses were performed with SPSS 22.0 for Windows.

## Results

**Fig. 1:**
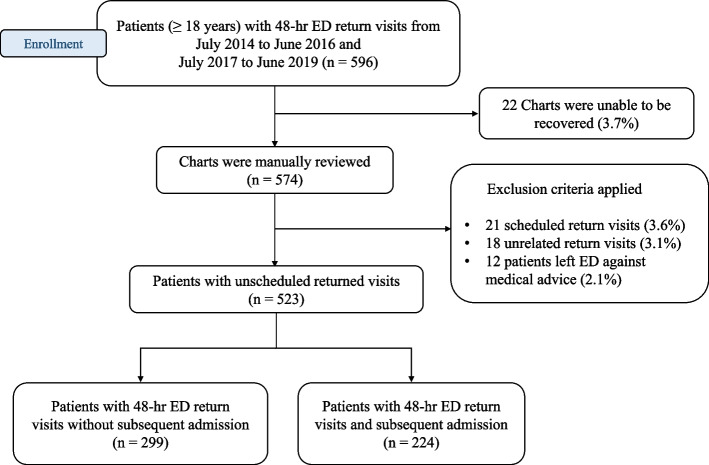
Flow diagram of the medical record data collection process

During a four-year study period between July 1, 2014, and June 30, 2016, as a preintervention period, and between July 1, 2017, and June 30, 2019, as a postintervention period, 165,405 patients presented to the ED. Of those visits, 596 (0.36%) patients returned to the ED within 48 h. Charts were available for review for 574 patients. Patients who revisited the ED within 48 h of initial discharge for unrelated medical problems, those with scheduled revisits, and those who left the ED against medical advice were excluded (Fig. 1). Of the 523 patients, 224 (43%) experienced subsequent admission to the hospital, and 299 (57%) patients were treated, evaluated, and released from the ED at the second visit.

**Fig. 2:**
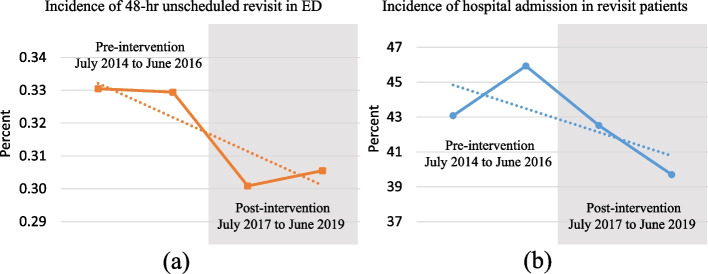
Time sequence plot and trend line of the pre- and postintervention periods show **a** the incidence of 48-h unscheduled return visits and **b** the incidence of subsequent hospital admissions among revisit patients, respectively

On average, 48-h unscheduled revisits occurred in 0.33% of all ED patients in the preintervention period and 0.30% of ED patients in the postintervention period, as shown in Fig. 2. The rate of hospital admission at the second ED visit decreased from 44.5 to 41.1% (95% CI of − 5.05 to 11.78) during the preintervention and postintervention periods. Table 2 shows the characteristics of the patients. There were no significant differences between the two periods in terms of the incidence of unscheduled return visits with subsequent admission, patient characteristics, or patient disposition at the second visit.

**Table 2:** Characteristics of 48-h unscheduled related return patients discharged from the ED

Characteristics	Preintervention period, % (*n* = 265)	Postintervention period, % (*n* = 258)	95% CI of the difference, %
Incidence of return visits with subsequent admission	44.5	41.1	− 5.05 to 11.78
**Age**			
15–30 years 31–45 years 45–60 years 61–75 years > 75 years	20.8 16.6 19.6 24.9 18.1	26.7 16.7 16.3 23.3 17.1	− 1.39 to 13.14 − 6.29 to 6.52 − 3.31 to 9.86 − 5.73 to 8.89 − 5.56 to 7.53
**Sex**			
Male Female	46 54	46.9 53.1	− 7.59 to 9.37 − 7.59 to 9.37
**Initial diagnosis category**			
Trauma Circulatory Respiratory Digestive Genitourinary Skin and subcutaneous tissue Infectious Musculoskeletal Neurological Others	3.8 5.7 19.2 26.8 11.7 3.0 10.9 1.1 6.0 11.7	8.1 3.0 10.1 19.4 15.5 2.7 20.2 3.9 8.1 7.0	0.20 to 8.6 − 0.92 to 6.48 3.03 to 15.13 0.16 to 14.52 − 2.10 to 9.74 − 2.84 to 3.44 3.09 to 15.51 0.03 to 6.00 − 2.37 to 6.68 − 0.34 to 9.79
**Length of stay in ED at 1st visit**			
< 24 h 24–48 h > 48 h	94.3 3.8 1.9	93.8 5.0 1.2	− 3.69 to 4.75 − 2.47 to 4.99 − 1.76 to 3.28
**ED visits within the past 12 months**			
0 1 > = 2	66.8 23.8 9.4	73.3 22.9 3.9	− 1.35 to 14.23 − 6.35 to 8.12 1.20 to 9.97
**Hospitalizations within the past 12 months**			
0 1 > = 2	68.7 23.4 7.9	75.2 19.4 5.4	− 1.20 to 14.08 − 3.05 to 10.98 − 1.88 to 6.94
**Charlson comorbidity index**			
0–1 > = 2	59.2 40.8	63.6 36.4	− 3.92 to 12.63 − 3.92 to 12.63
**Severity based on 1st visit triage**			
ESI V (white) ESI IV (green) ESI III (yellow) ESI II (orange)	6.4 15.5 66.8 11.3	3.9 12.8 71.7 11.6	− 1.41 to 6.52 − 3.32 to 8.70 − 3.00 to 12.71 − 5.21 to 5.84
**Patient deposition at 2nd visit**			
Discharge home Observation unit Hospital floor Refer Critical care unit	50.2 5.3 38.5 2.6 3.4	53.9 5.0 34.9 3.5 2.7	− 4.83 to 12.15 − 3.68 to 4.26 − 4.64 to 11.76 − 2.25 to 4.19 − 2.50 to 3.94

*p value < 0.05

*ESI* Emergency Severity Index

Patient characteristics were compared between patients who were discharged from the ED and those who required hospital admission at the second visit (Table 3). The latter group had a significantly higher comorbidity score, a higher number of ED visits, and a higher number of hospitalizations within the past 12 months. We found that two patient age groups (61–75 years and > 75 years) had an increased risk of subsequent hospital admission compared with younger patients. Of the initial diagnosis categories, the highest difference in return visits and the need for hospital admission was found in the infectious category. A significantly higher hospital admission rate was observed in patients with a higher triage score (ESI II) at their first visit, while patients with lower triage scores (ESI III, IV, V) had a lower rate of subsequent hospital admission.

**Table 3:** Characteristics of the discharged and admitted patients at the second ED return visit

Characteristics	Revisit with subsequent admission, % (*n* = 224)	Revisit then discharge, % (*n* = 299)	95% CI of the difference, %
**Period**			
Preintervention Postintervention	52.7 47.3	49.2 50.9	− 5.12 to 12.04 − 5.02 to 12.14
**Age**			
15–30 years 31–45 years 45–60 years 61–75 years > 75 years	20.1 10.7 15.2 29.9 24.1	25.8 21.1 20.1 20.4 12.7	− 1.66 to 12.75 4.04 to 16.43* − 1.82 to 11.30 2.02 to 17.05* 4.73 to 18.26*
**Sex**			
Male Female	43.3 56.7	48.8 51.2	− 3.12 to 13.97 − 5.36 to 11.73
**Initial diagnosis category**			
Trauma Circulatory Respiratory Digestive Genitourinary Skin and subcutaneous tissue Infectious Musculoskeletal Neurological Others	2.2 6.3 17.4 26.3 9.8 1.3 22.8 0.0 6.7 7.1	8.7 4.7 12.7 20.7 16.4 4.0 10.0 4.3 7.4 11.0	0.32 to 7.75* − 2.32 to 5.99 − 3.81 to 8.90 − 4.35 to 10.48 0.65 to 12.24* − 0.32 to 5.69 6.43 to 19.40* 0.62 to 5.48* − 4.04 to 5.08 − 1.24 to 8.76
**Length of stay in ED at 1st visit**			
< 24 h 24–48 h > 48 h	92.9 4.5 2.7	95.0 4.3 0.7	− 1.99 to 6.67 − 3.37 to 4.18 − 0.27 to 5.08
**ED visits within the past 12 months**			
0 1 > = 2	59.8 29.5 10.7	77.6 18.7 3.7	9.79 to 25.65* 3.43 to 18.25* 2.59 to 11.99*
**Hospitalizations within the past 12 months**			
0 1 > = 2	60.3 30.8 8.9	80.6 14.4 5.0	12.43 to 28.00* 9.19 to 23.64* − 0.45 to 8.76
**Charlson comorbidity index**			
0–1 > = 2	54.9 45.1	66.2 33.8	2.85 to 19.61* 2.85 to 19.61*
**Severity based on 1st visit triage**			
ESI V (white) ESI IV (green) ESI III (yellow) ESI II (orange)	3.6 8.9 66.5 20.9	6.4 18.1 71.2 4.3	− 1.39 to 6.36 3.24 to 14.86* − 3.25 to 12.73 10.96 to 22.65*

*p value < 0.05

*ESI* Emergency Severity Index

The crude odds ratio (OR) for the association showed lower odds for hospital admission at the second visit in the postintervention period, but this difference was not statistically significant (OR crude = 0.87; 95% CI 0.61 to 1.23, Table 4). Therefore, this analysis reflected a small effect attributable to the study period and the intervention on the incidence of return visits with subsequent admission. The multivariate analysis of the case–control subgroup suggested that a length of stay > 48 h at the index ED visit, having more ED visits or hospitalizations within the past 12 months, chronic conditions, and higher triage severity increased the risk of return visits with subsequent admission. Older patients (> 60 years) had higher odds of returning and requiring admission than younger people, with the odds increasing with age. Patients with infectious diseases still had the highest odds of return admission among the other initial diagnostic categories. The most common final diagnosis from this category was acute febrile illness (Supplementary Table S1).

**Table 4:** Multiple logistic regression models of unscheduled related return visits after discharge from the ED

Characteristics	Revisit with subsequent admission (*n* = 224)	Revisit then discharge (*n* = 299)	Odds ratio (95% CI)
**Period**			
Preintervention Postintervention	118 106	147 152	Reference 0.87 [0.61 to 1.23]
**Age**			
15–30 years 31–45 years 45–60 years 61–75 years > 75 years	45 24 34 67 54	77 63 60 61 38	Reference 0.65 [0.36 to 1.12] 0.97 [0.55 to 1.69] 1.88 [1.13 to 3.12]* 2.43 [1.38 to 4.23]*
**Sex**			
Male Female	97 127	146 153	Reference 1.25 [0.88 to 1.77]
**Initial diagnosis category**			
Trauma Circulatory Respiratory Digestive Genitourinary Skin and subcutaneous tissue Infectious Musculoskeletal Neurological Others	5 14 39 59 22 3 51 0 15 16	26 14 38 62 49 12 30 13 22 33	Reference 5.20 [1.55 to 17.44]* 5.34 [1.86 to 15.35]* 4.95 [1.78 to 13.74]* 2.33 [0.79 to 6.88] 1.30 [0.27 to 6.35] 8.84 [3.07 to 25.46]* - 3.54 [1.11 to 11.32]* 2.52 [0.82 to 7.79]
**Length of stay in ED at 1st visit**			
< 24 h 24–48 h > 48 h	208 10 6	284 13 2	Reference 1.05 [0.45 to 2.44] 4.09 [0.82 to 20.49]
**ED visits within the past 12 months**			
0 1 > = 2	134 66 24	232 56 11	Reference 2.04 [1.35 to 3.09]* 3.77 [1.79 to 7.95]*
**Hospitalizations within the past 12 months**			
0 1 > = 2	135 69 20	241 43 15	Reference 2.86 [1.85 to 4.43]* 2.38 [1.18 to 4.80]*
**Charlson comorbidity index**			
0–1 > = 2	123 101	198 101	Reference 1.61 [1.13 to 2.29]*
**Severity based on 1st visit triage**			
ESI V (white) ESI IV (green) ESI III (yellow) ESI II (orange)	8 20 149 47	19 54 213 13	1.14 [0.43 to 3.01] Reference 1.89 [1.08 to 3.29]* 9.76 [4.38 to 21.73]*

*p value < 0.05

*ESI* Emergency Severity Index

## Discussion

ED overcrowding is considered a complex problem, and the need to reduce or eliminate it has received considerable attention [24]. Various studies have proposed effective strategies to reduce ED readmission and investigated their effectiveness in terms of the quality of care delivered to patients [25]. One Canadian study was designed to evaluate the effect of interventions to decrease ED crowding including increased ED physician coverage, the designation of physician coordinators, and new hospital policies [26]. Their results showed no difference in the incidence of return visits although the mean length of stay for patients in the ED was reduced. Our intervention in increasing medical staff, although we did not implement discharge planning or other medical interventions, did not significantly decrease the incidence of ED revisit admissions, and the ED length of stay was not influenced (Table 2).

The quality of medical attention that a patient receives in the ED has also been reported as a significant factor related to the incidence of return visits [10]. The study from Tsai et al. showed the highest prevalence of patients with return visits and intensive care unit (ICU) admissions during the evening shift, which was associated with the impact of ED crowding and the number of available physicians [22]. The 0.36% incidence of ED revisits in this study was less than that reported by most studies (approximately 3%) [23, 27, 28], which can be explained by their shorter 48-h revisit window, while our hospitals used EDs as admission holding spaces and for the care of all injury cases seen in the emergency room (ER). Following our intervention of increasing the number of ED physician, we observed a small decline in both overall ED return visit rates and the rate of subsequent hospital admissions. Previous studies also observed an improvement in ED flow efficiency and reduced ED waiting times with senior physician involvement in patient evaluation and treatment [29, 30]. Senior physician initial assessment can speed up treatment for all patients through good planning and appropriate use of investigations [30]. In our study, there could not be an increase in the additional number of ED attending staff during the intervention period, which could explain the nonsignificant impact of our findings.

There are multiple explanations as to why only increasing physician coverage did not help with the incidence of revisits. The ED is a multidisciplinary environment, and patients are complex. Although diagnostic workup and risk stratification are the primary focus in evaluating patients, physicians can encourage overdiagnosis and overtreatment, introducing additional risks. Effective discharge planning is crucial for effective continuing care, and suboptimal discharge instruction was associated with an increased rate of ED return visits as were demonstrated by some studies [31–33]. Educational intervention to ensure physician knowledge and skills regarding patients’ better health outcomes and optimal management will help avoid redundant or unnecessary use of ED diagnostics and resources [34].

The most noteworthy finding of our study was the higher rate of revisit admissions among high ED users defined as those with 2 or more ED visits in a 12-month period, patients with prior hospitalizations within 1 year, and patients with serious comorbidities. Older age was a risk factor in both our study and a previous study by Martin-Gill and Reiser [35], especially those with comorbidities, who were at higher risk for revisit admissions. Another work published by Fan et al. [36] showed that an age of 65 years or older and multiple comorbidities were risk factors for unexpected ICU admission after discharge from the ED. High ED users and patients with prior recent hospitalizations are higher severity patients, and physicians would expect a concurrent increase in the admission rate [37]. Our results imply that the patient's underlying condition, including age and comorbidities, are the major predictors of ED revisit admission.

An increased rate of revisits and admissions was also found in the higher triage severity level determined by the ESI at the patients’ first visit; this was especially common in the ESI level II group. This finding is consistent with the above findings regarding the severity of a patient, which has been the most often isolated risk factor for hospital readmission [11]. On the other hand, the study by Gao et al. [38]. showed that the number of prior-year hospitalizations was correlated with ED revisits. Without knowing the exact reason(s), we hypothesize that these variables are indicators of disease severity that warrants inpatient rather than ED care [39], and higher mortality may also play a role.

The diagnostic category resulting in the highest rate of hospital admissions was infectious diseases (Supplementary Table S1). The most common primary diagnosis at the second visit in this disease category was unspecified fever (74%), followed by unspecified acute febrile illness (28%). In multiple previous studies, fever was one of the most common initial ED presentations among ED revisit patients [11, 27, 40]. ED patients with unexplained fever frequently show no localized symptoms or signs to suggest a fever source [41]. Gur et al. [42] reported that the etiology of unexplained fever in the admission and discharge groups combined was still unknown in 71.22% of cases after a 30-day follow-up. Obtaining a definitive diagnosis could be difficult, as 76% of our revisit-discharged patients still did not have a definitive diagnosis (Supplementary Table S1). This finding also suggested that establishing a protocol to prevent pitfalls in managing patients with fever is imperative to decrease ED revisits. Finally, we noticed that the second subsequent ED admission was not correlated with the length of stay at the initial ED visit.

There are multiple factors that could be involved in the quality of patient care. A protective mechanism to prevent unexpected hospital revisits and admissions should be constructed and implemented in the treatment of patients. The studies conducted to date to identify patients with the highest risk of ED readmission have had mixed results [25, 43, 44]. The study by Abulalenain et al. on 72-h ED return admissions found that the quality issues from the previous visits and poor outcome problems were rare on the second ED visit [45]. However, conducting a study regarding return admissions without a good review process may not be a good way to measure clinical quality. An important agreement from those studies is that the care provided to ED patients must go beyond treating the disease and paying attention to implement a useful tool to ensure patient-centered care and avoid prevention efforts that mistakenly focus on any specific factors.

## Limitations

The study was a retrospective study carried out in a single tertiary hospital, and therefore, the results may not be generalizable to other settings. Furthermore, the availability of insufficient data was difficult to overcome. Nevertheless, the effect of insufficient data was reduced to a minimal level, as the data we collected were the basic information required to complete the routine care of our patients. The identified risk factors for unexpected hospital admissions could be relevant only to increases in the occurrence and cannot be recognized as independent risk factors. We did not enroll patients who may have revisited other hospitals, those who were admitted 2 days after ED discharge, or those who died before the return visit, which might result in an underestimation of the occurrence of severe undesired events of ED management in general. Hence, the severity and overall revisit rate of the study population tended to be lower than those in previous studies.

## Conclusion

In our study, no association was found between our intervention of increasing ED physician and the rate of ED revisit admissions. Some factors we identified in this study seem to have some benefits and can be used to prevent 48-h ED revisit admissions. Patient-centered care is one of the core goals of emergency care, and further efforts to develop interventions to improve the quality of care provided to ED patients are warranted.

## Supplementary Information

**Additional file 1.** 

## Data Availability

The relevant data used to support the findings of this study are available from the corresponding author upon request.
