# Supplementary material for: Reducing 48-h emergency department revisits and subsequent admissions: a retrospective study of increased emergency medicine resident floor coverage
Source: Int J Emerg Med. 2022 Dec 6;15:66. doi: 10.1186/s12245-022-00471-z (PMC9724369; doi:10.1186/s12245-022-00471-z)
Supplement: Supplementary file 1 — Additional file 1. [file 12245_2022_471_MOESM1_ESM.docx]

**Supplementary Material**


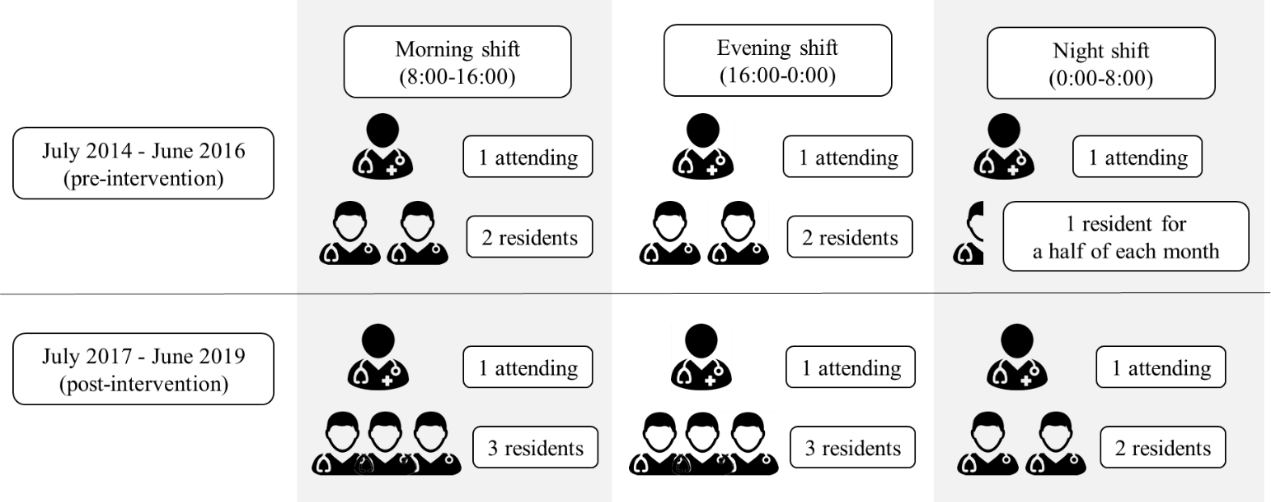


Figure S1. Graphic depicting the intervention, which consisted of 24-hour emergency medicine (EM) resident coverage and more EM residents per shift.

* Both the pre- and postintervention periods had the same number of rotating residents from other specialties.

| Primary diagnoses (n=81) |
| --- |
| Fever, unspecified (38) |
| Acute febrile illness, unspecified (23) |
| Dengue (10) |
| Viral infection, unspecified (4) |

Final diagnoses

| Revisit with subsequent admission (n=51) | Revisit followed by discharge (n=30) |
| --- | --- |
| Urinary tract infection (9) | Acute febrile illness, unspecified (10) |
| Tropical infection* (8) | Viral infection, unspecified (7) |
| Dengue (7) | Fever, unspecified (6) |
| Influenza (5) | Urinary tract infection (3) |
| Acute cholecystitis (4) | Tropical infection* (2) |
| Primary bacteremia (4) | Dengue (2) |
| Acute febrile illness, unspecified (4) |  |
| Malaria (3) |  |
| Viral infection, unspecified (3) |  |
| Fever of unknown origin (2) |  |
| Melioidosis (1) |  |
| Acute appendicitis (1) |  |

* Leptospirosis, scrub typhus, rickettsia.

Table S1. Primary diagnoses and final diagnoses of ED return visit patients by infectious disease category (n=81)
